# Bioactive Carbohydrate Polymers—Between Myth and Reality

**DOI:** 10.3390/molecules26237068

**Published:** 2021-11-23

**Authors:** Maroua Drira, Faiez Hentati, Olga Babich, Stanislas Sukhikh, Viktoria Larina, Sana Sharifian, Ahmad Homai, Imen Fendri, Marco F. L. Lemos, Carina Félix, Rafael Félix, Slim Abdelkafi, Philippe Michaud

**Affiliations:** 1 Laboratoire de Biotechnologies des Plantes Appliquées à l’Amélioration des Cultures, Faculté des Sciences de Sfax, Université de Sfax, Sfax 3038, Tunisia; maroua.drira.etud@fss.usf.tn (M.D.); imen.fendri@fss.usf.tn (I.F.); 2 INRAE, URAFPA, Université de Lorraine, F-54000 Nancy, France; faizhentati@gmail.com; 3 Institute of Living Systems, Immanuel Kant Baltic Federal University, A. Nevskogo Street 14, 236016 Kaliningrad, Russia; olich.43@mail.ru (O.B.); stas-asp@mail.ru (S.S.); surinac@mail.ru (V.L.); 4 Department of Marine Biology, Faculty of Marine Science and Technology, University of Hormozgan, Bandar Abbas 74576, Iran; sharifian_sana@yahoo.com (S.S.); a.homaei@gmail.com (A.H.); 5 MARE–Marine and Environmental Sciences Centre, ESTM, Polytechnic of Leiria, 2520-641 Peniche, Portugal; marco.lemos@ipleiria.pt (M.F.L.L.); carina.r.felix@ipleiria.pt (C.F.); rafael.felix@ipleiria.pt (R.F.); 6 Laboratoire de Génie Enzymatique et Microbiologie, Equipe de Biotechnologie des Algues, Ecole Nationale d’Ingénieurs de Sfax, Université de Sfax, Sfax 3038, Tunisia; slim.abdelkafi@enis.tn; 7 Université Clermont Auvergne, CNRS, Clermont Auvergne INP, Institut Pascal, F-63000 Clermont-Ferrand, France

**Keywords:** polysaccharide, oligosaccharide, bioactive agent, macromolecules

## Abstract

Polysaccharides are complex macromolecules long regarded as energetic storage resources or as components of plant and fungal cell walls. They have also been described as plant mucilages or microbial exopolysaccharides. The development of glycosciences has led to a partial and difficult deciphering of their other biological functions in living organisms. The objectives of glycobiochemistry and glycobiology are currently to correlate some structural features of polysaccharides with some biological responses in the producing organisms or in another one. In this context, the literature focusing on bioactive polysaccharides has increased exponentially during the last two decades, being sometimes very optimistic for some new applications of bioactive polysaccharides, notably in the medical field. Therefore, this review aims to examine bioactive polysaccharide, taking a critical look of the different biological activities reported by authors and the reality of the market. It focuses also on the chemical, biochemical, enzymatic, and physical modifications of these biopolymers to optimize their potential as bioactive agents.

## 1. Introduction—What Is a Bioactive Polysaccharide?

Polysaccharides are biopolymers of carbohydrates commonly found in all living organisms. Polysaccharides are probably among the more complex macromolecules existing in nature. As proteins or nucleic acids, they are biopolymers; however, contrary to them including, respectively, in their structures 4 nucleotides (nucleic acids) or 20 amino acids (proteins), up to 40–50 monosaccharides (mainly pentoses and hexoses) have been detected in polysaccharides. These pentoses and hexose have the ability to link each other by glycosidic bonds between the anomeric hydroxyl of one monosaccharide with any of the other ones from another unit. Therefore, with the sole example of the assembly of two monosaccharides such as glucose and galactose, 12 putative glycosidic bonds are potentially possible, leading to various disaccharides. Most polysaccharides found in nature occur as medium or high molecular weight biopolymers, including in their structure’s numerous monosaccharides. Also called glycans, they differ by the length of their chains, the monosaccharides units composing them, and also the type of glycosiding linkages in their structures and the degree of branching leading to linear or ramified polysaccharides. Homopolysaccharide contain only a single type of repeating unit, whereas heteropolysaccharides contain two or more different monosaccharide kinds. This high level of variability is reinforced by non-osidic chemical groups grafting the polysaccharidic backbone such as short organic acids (acetyl, pyruvyl, or succinyl groups) or sulfuric acid half ester (mainly in marine polysaccharides) [1,2,3]. The physiological functions of polysaccharides are diverse and are acquired after forming specific conformations. They are also strongly dependent on linkage modes functions and properties appearing accordingly. Some of them are carbon and energy storage macromolecules such as starch, glycogen, or laminaran. Others are structural elements, as in the case of the chitin of exoskeletons of some animals and fungi or cellulose in plants. More recently, some polysaccharides have been also identified as bioactive compounds and called bioactive polysaccharides. This definition refers to polysaccharides having biological effects on living organisms. Indeed, these biopolymers participate in many biological processes, notably cellular communication. Glycosciences have focused more and more on the identification of the native biological functions of some bioactive polysaccharides, as well as on the use and screening of others with no natural functions, with the objective of bringing them to market. This review detailed the natural or modified bioactive polysaccharides showing nutraceutic, therapeutic or toxic actions on microorganisms, animals, and plants. These biological activities are strongly affected by the chemical structures of polysaccharides, and the reviews are the state-of-the-art of recent progress in their functionalization of using physic, chemistry, or biochemistry, and aim to correlate some biological activities to structural features. Moreover, in front of the exponential increase in articles, for book chapters and articles dealing with new bioactive polysaccharide with promising industrial applications in various fields, notably in medicine, a special attention is given to the market reality. However, even if some polysaccharides have already found various applications as bioactive compounds in the therapeutic fields as an anticoagulant agent (heparin); for instance, hydrogels (hyaluronic acid), or in vaccines notably after conjugation with a protein carrier, have their development limited to niche markets such as those of nutraceutic (prebiotics and synbiotics) and cosmetics. However, exploration in other fields such those of plant elicitation and biostimulation, wound healing, drug delivery, or tissue engineering using notably 3D printing could exist in the future. The recent exploration of a new potential source of polysaccharides such as those produced by microalgae opens the way to a new structure of high potential.

## 2. Structure-Function Relationships

Polysaccharides perform essential biological functions in the human body, such as antioxidant, immunomodulatory, antitumor, etc., [2,4,5,6,7,8,9,10,11,12]. However, these properties are strongly correlated with the carbohydrate polymer structure: its molecular weight, monosaccharide composition, type of glycosidic bond, and the degree of chain branching [3,13,14,15,16].

### 2.1. Antioxidant Function of Polysaccharides

The radical cation 2,2′-Azino-bis (3-ethylbenzthiazoline-6-sulfonic acid) (ABTS) is often used to evaluate the general antioxidant activity of compounds [17]. Zhang et al. [18] showed that plant polysaccharides with higher mannose content and lower glucose content exhibit more pronounced scavenging activity against free radicals ABTS. Uronic acid with electrophilic groups (such as ketonic or aldehyde) in the acidic polysaccharide promotes the release of hydrogen from the OH bond [19]. Moreover, low molecular weight polysaccharides had more reductive hydroxyl ends (per unit weight) to accept and eliminate free radicals [18]. The activity of polysaccharides in scavenging ABTS radicals depends on the content of protein, uronic acid residues, and glucose [14,18,20,21].

There is a relationship between the average molecular weight of the polymer and the ability to scavenge 2,2-diphenyl-1-picrylhydrazyl (DPPH) radicals. The lower the molecular weight is, the higher the antioxidant activity relative to DPPH [14,18]. It can be assumed that the value of the antioxidant activity is also influenced by the content of mannose, rhamnose, uronic acid, and protein components in the polymer composition [14,20,22,23]. Zhang et al. [18] demonstrated a significant correlation of activity within the content of arabinose and galactose. It is reported that the ability to scavenge DPPH radicals of neutral polysaccharides is greater than that of acid [24].

The scavenging activity of hydroxyl radicals increases with increasing concentration. Polysaccharides with a high content of uronic acid, mannose, and protein components have a higher antioxidant activity [14,25,26,27]. Zhang et al. [18] showed that a polysaccharide with a higher content of uronic acid and rhamnose and a lower glucose content exhibited the highest absorbing activity.

The activity of scavenging superoxide radicals depends on the content of uronic acid residues and protein components, as well as on the monosaccharide composition of the backbone [14,18,25]. High scavenging activity was exhibited by a heteropolysaccharide, which contained a high content of uronic acid and, consequently, a high content of carboxyl groups, which weakened the dissociation energy of the O–H bond, which led to higher scavenging activity [18]. A higher antioxidant activity against superoxide radicals was observed for samples with a large amount of protein components in the polysaccharide composition [14].

Zeng et al. [28] and Wang et al. [29] demonstrated that acidic polysaccharides, in which the monomeric units of the backbone are connected mainly by a β-glycosidic bond, had antioxidant activity. However, in Rozi et al. [30], the acidic polysaccharide, which had only α-glycosidic bonds in its structure, had the highest antioxidant activity. This can be explained by the fact that these samples differed significantly in their weight-average molecular weight, which had a significant effect on the activity of the polymers.

With an increase in concentration to a certain value, the antioxidant activity increased, but the activity may decrease when this concentration value is exceeded [22,23,26,31]. The absorption capacity for metal ions increased with an increase in the polysaccharide concentration [20,22,23,29,32]. The galactose content also showed a significant correlation with the reducing ability of ferrous iron ions [33].

The xylose content had a significant effect on the chelating activity of metals [33]. It was shown in [29] that the neutral polysaccharide had a significantly higher chelating activity of Fe^2+^ than acidic polysaccharides.

An increase in the polysaccharide concentration also enhanced the effect of inhibiting the discoloration of β-carotene [22,25]. Polysaccharides, which mainly contained residues of mannose, ribose, glucose, galactose, xylose, and arabinose, had the potential for lipid peroxidation [22].

Thus, many polysaccharides are effective antioxidants. However, the mechanism is still not clear enough, and the relationship between the structure of the polysaccharide and its scavenging capacity has not been fully elucidated due to the wide variety and variability of the structural features of the molecules of these polymers.

### 2.2. Immunomodulatory Function of Polysaccharides

Plant polysaccharides are ideal candidates for immunomodulatory therapeutic agents due to their relatively low toxicity [18,34,35,36,37,38,39]. They can improve the viability of macrophage cells. Polysaccharides with a high proportion of galactose residues in their structure significantly promoted the proliferation of RAW264.7 cells [40,41,42]. However, a high concentration of carbohydrate polymers can also have the opposite effect [18,43].

Polysaccharides with higher molecular weights better activate NO production [18,35]. The activation of macrophages by plant polysaccharides is mediated by recognizing polysaccharide polymers by specific receptors [44]. High molecular weight polysaccharides can have even more repetitive receptor sites that can recognize receptors on macrophages [18]. Many polysaccharides exhibiting immunomodulatory activity contain significant amounts of mannose and galactose residues in their structure [18,29,35,45]. Sulfated polysaccharides have a high potential for immunological activity [32,46]. The NO production in macrophages increased in a concentration-dependent manner upon treatment with various concentrations of the polysaccharide [25,36,43].

High molecular weight polysaccharides also activate cytokine production [34]. Pectin fractions with a large amount of galacturonic acid led to a higher production of IL-1α and G-CSF cytokines by murine macrophages compared to other fractions [47]. The proliferation of splenocytes was influenced by polysaccharides with high molecular weights and containing residues of mannose or galactose [25,43]. Polysaccharides containing d-glucose and d-mannose residues in a certain concentration had a protective effect against the apoptosis of macrophages caused by H_2_O_2_ [22,43].

The immunomodulatory activity of polysaccharides, determined in various studies, is summarized in Table 1.

### 2.3. Antitumor Function of Polysaccharides

Antitumor activity against cancer cells depends on the structure of the polysaccharide backbone [14]. Most polymers with such activity contain such monosaccharide residues as glucose and mannose [17,20,21,23,48]. However, the action of polysaccharides is specific for various cancer cells [17,20]. The concentration dependence of the antiproliferative activity of polysaccharides was established [14,21,23,49,50]. A higher degree of branching led to stronger in vitro antitumor activity [51].

Thus, polysaccharides have different structural features, such as monosaccharide composition, type of glycosidic bonds, average molecular weight, degree of branching, presence or absence of protein components, or any functional groups. At the same time, the influence of various structural features on biological activity are interconnected with each other, therefore they must be considered in conjunction.

## 3. Elicitation and Biostimulation

From an agronomic perspective, elicitor treatments with polysaccharides have gained prominence since their potential not only for modern agriculture practice but also as alternative tools to agrochemicals, allowing us to solve some environmental damage [52]. In the last decades, poly or oligosaccharides derived from algae have been more focus due to their ability to activate plant signals that enhance secondary metabolites’ production and trigger plant defense responses [53,54]. Given their great diversity, these compounds have been also suggested to act as plant growth stimulators, which aim to intensify the absorption of substances or nutrients that will reduce chemical fertilizers [55].

**Figure 1:**
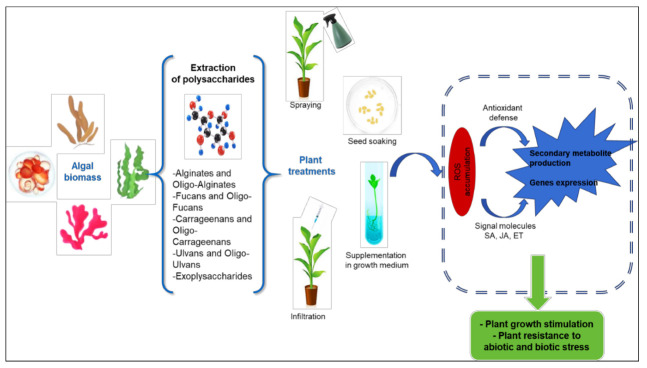
Schematic representation of the general immune response of plants under algae polysaccharides treatments. SA: Salicylic acid; JA: Jasmonic acid; ET: Ethylene.

Although it is largely established that polysaccharides act as effective biotechnological tools, their strategies’ effects are not generalized. In fact, plant metabolism depends on the study model, characteristics of polysaccharides (structure, molecular mass, degree of polymerization and sulfate content) and types of treatment, e.g., foliar spraying, seed soaking or supplementation on growth medium (Figure 1).

In recent years, tremendous advances have been made in understanding the general mode of action of algae polysaccharides as elicitors; however, their structure–activity relationships are still not well known.

Below, we briefly summarize the main results of the studied effects of poly- and oligosaccharides on plants’ immune systems provided in the literature.

### 3.1. Polysaccharides as Inducers of Plant Defenses

The induction of resistance in plants by macroalgae polysaccharides is an effective method for disease control against environmental constraints which is linked to the activation or enhancement of several plant defense mechanisms such as the positive regulation of the expression of defense genes involved in the response of a wide range of abiotic and biotic stress, and the induction of the antioxidant system and the production of secondary metabolites [54,56,57,58]. Alginate or alginate oligosaccharides are the most algae carbohydrates described as potent elicitors in the literature (Table 2). For example, spray treatment with alginate in tomato induced the temporary production of reactive oxygen species, activated antioxidant defense, and increased the expression level of genes belonging to the SA-, JA-, and ET pathways conferring growth inhibition of *Alternaria solani* [59]. In addition, alginates of *B. bifurcata* and *F. spiralis* were shown to stimulate natural defenses of date palm by the activation of PAL activity and phenolic compound production [60]. Likewise, foliar application of κ-carrageenan in tomatoes reduced the severity of leaf spot caused by *Septoria lycopersici* via activation of antioxidant defense and modulation of chloroplast proteome [61]. Similarly, Tobacco infiltration with sulfated-carrageenan induced resistance to tobacco mosaic virus through the over-expression of defenses related genes encoding to PR proteins [62]. Sangha et al. [63] reported that only sulfated carrageenans have the ability to enhance resistance to *Trichoplusia ni* in *Arabidopsis thaliana*, indicating that the jasmonic acid and salicylic acid pathways are involved in this resistance. Fucan was also demonstrated to induce a multiplicity of plant defense events [54]. Klarzynski et al. [64] observed that fucan induced tobacco resistance to tobacco mosaic virus via the activation of some markers of systemic acquired resistance (SAR), such as the accumulation of salicylic acid and expression of the PR1 gene. Interestingly, and in the same way, microalgae polysaccharides were proved to trigger plant defense responses. Rachidi et al. [65] reported that polysaccharides extract from six different microalgae and cyanobacteria induced multiple signaling pathways in tomato, such as accumulation of ROS, pathogenesis-related proteins, and fatty acids, and improves the PAL and POX activities. Recently, Drira et al. [66] proved the potential of exopolysaccharides produced by *Porphyridium sordidum* to attenuate the severity of *Fusarium oxysorum* in *Arabidpsis thaliana* leaves. These authors provided evidence that EPS foliar applications activate the production of H_2_O_2_ and enhance defense-related marker gene activities such as PAL, SOD, POD, CYP, and PR1.

### 3.2. Polysaccharides as Plant Growth Stimulator

In addition to their capacity to induce a defense response, algae polysaccharides can also stimulate plant growth. In fact, the growth promotion effect of polysaccharides extracted from microalgae [73,74,75] and macroalgae [55] was clearly demonstrated in terms of plant weight, plant size, leaves number, root length, chlorophyll content and photosynthetic activity. The biostimulant effect of purified poly or oligosaccharides has also been supported by several studies. For example, Yang et al. [76] have shown that the soaking of barley seeds in solutions of alginate-derived oligosaccharides enhanced seedling growth. This growth enhancement, which is linked to the molecular weights and Mannuronate/Guluronate ratio, was promoted by the stimulation on photosynthesis and amelioration of the adsorption activity. Similarly, *Eucomis autumnalis* bulbe coating by a low molecular mass oligoalginate increases plant height, florets number per inflorescence, and relative chlorophyll content [77]. In addition, foliar tobacco application by oligo carrageenans increases leaf biomass by stimulation photosynthesis efficiency, ribulose 1,5-biphosphate carboxylase/oxygenase, and NAD(P)H-synthesizing enzymes activities [78]. Oligo carrageenans were also shown to enhance the level of growth-promoting hormones and C, N, and S assimilation in pine trees [79].

With increasing data from diverse research, algae polysaccharides appear to be an amazingly versatile elicitor agent. However, the potential interest of their commercializing is still in the early stages for agricultural use.

## 4. Medicine

The potential of marine natural resources and recent advances in marine medicine biotechnology have led to the recent scale-up of the vast applications of marine resources in the medical area. Among a myriad of resources, recently, marine polysaccharides extracted from macro- and microalgae and bacteria have attracted the attention researchers worldwide [80]. The biocompatibility, biodegradability, adhesivity, diversity of chemical structures, low toxicity, and the ability to form hydrogels in marine polysaccharides [4,81,82,83] resulted in their vast use not only in food and cosmetic industries, but also led to their applications as materials for the incorporation of bioactive agents in drug delivery systems [84,85,86,87,88,89,90,91,92].

Marine sulfated polysaccharides including carrageenan, ulvan, and fucoidan extracted from macro algae or seaweeds [93,94,95,96], as well as microbial exopolysaccharides produced by extreme bacteria, fungi, and microalgae [97,98] are marine polysaccharides frequently reported during recent years because of their numerous biological properties, including antioxidant, anticoagulant, anticancer, antiviral, antiallergic, antiadhesive, antiangiogenic, and anti-inflammatory actions, as well as their high potential for deliver drug systems and tissue engineering [99,100,101,102,103,104,105,106,107]. New chemical modification methods have been developed during recent years to improve some of the biological activities of sulfated polysaccharides, to change their affinity to specific drugs, to increase their ability to incorporate drugs, and, finally, to increase the efficacy of their release [83,108].

**Figure 2:**
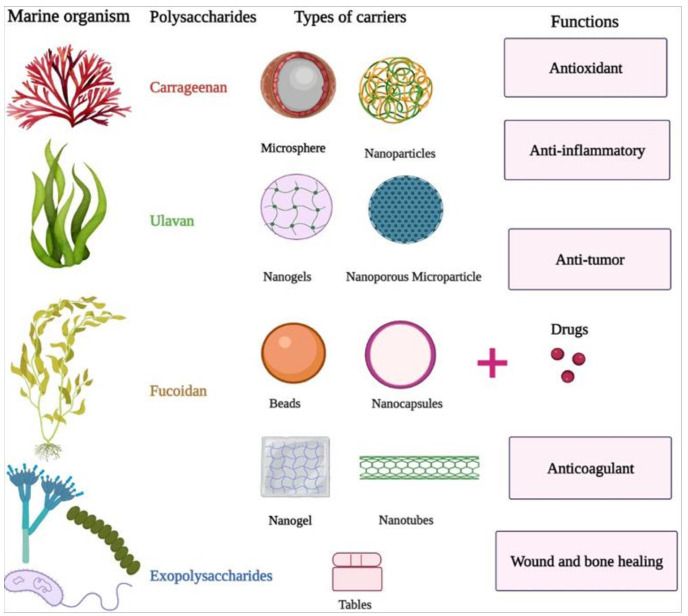
The diversity of polysaccharides extracted and carriers developed from marine sources and potential pharmaceutical functions.

Recently, in variety of carriers containing marine polysaccharides have been developed including nanoparticles, nanogels, nanotubes, nanocapsules, nanoporous microparticles, microspheres, hydrogels, beads, and tables, which effectively deliver drugs to target tissue in drug delivery systems [90,109,110,111,112,113,114,115,116,117,118,119] for medicine applications including gene therapy, cell therapy, pharmacokinetics, and tissue engineering (Figure 2). Here, we briefly review prominent developments of some dominant marine polysaccharides in deliver drug systems and tissue engineering.

κ-carrageenan, λ-carrageenan, and ι–carrageenan are three types of sulfated polysaccharides extracted from red algae [120] with the ability to form hydrogel for κ-carrageenan and ι–carrageenan [121]. Microscale fibers, beads, nanotubes, spheres, nanoparticles containing carrageenan have been developed for diverse biomedical applications [88,122,123] (Figure 2). Some of the biological activities reported from carrageenan include antioxidant [124], antitumor, and immunomodulation activities [125], as well as reducing serum cholesterol and triglyceride levels [126], regulating growth factors [127,128], inhibiting syncytium formation [129], and promoting function HaLa cell and fibloblasts for healing wound [130].

In the field of tissue engineering, the carrageenans have been frequently used to deliver growth factors [118,119], to immobilize enzymes [131], for healing wounds [132], and to encapsulate several cell types, e.g., with human-adipose-derived stem cells, human nasal chondrocytes, or chondrocytic cells to deliver them in vivo for cell therapies and cartilage regeneration [119,120,133,134,135]. κ and ι –carrageenan are potential carriers for the controlled release of drugs [136,137,138,139]. Carrageenan-based hydrogels, beads and nanoparticles have been developed to deliver albumin [109,110,119], rosmarinic acid (RA) [140], acyclovir (antiviral drug) [5], ibuprofen [85], insulin aspart [87], as well as for their usage as a cell carrier [88] and echinochrome A [89].

Ulvan is sulfated polysaccharides extracted from the green algae with more diverse and complex structures, as well as biological activities less studied in compared to other sulfated polysaccharides [141,142,143,144,145,146]. Up to now, the diverse forms of carriers using ulvan including nanofibers, membranes, particles, hydrogels, and 3D porous structures have been developed (Figure 2) for delivery drugs, for peptide/protein [147,148,149,150,151,152,153,154], for wound dressing or bone tissue engineering [147,148,149,150,151,155,156,157,158,159], for inhibiting HeLa [160] and glioblastoma cells [161], for proliferation splenocyte [162], for enhancing fibroblasts growth and angiogenesis [163], for enhancing differentiation PC-12 cells [164], and for proliferation mesenchymal stem cells [165].

Fucoidan, constituting 5–10% of the dry biomass of brown seaweeds is sulfated polysaccharide with a high diversity of structure [166,167]. The ability of fucoidan to form gels and films is far less compared to carrageenan and ulvan [168], thus the mixing of fucoidan with other polymers can result in gels and films [169,170]. Among biological activities, the anticoagulant property of fucoidan has been well known [171,172,173]. Moreover, up to now, other activities such as antiviral [174], antimetastasis and antilymphangiogenesis [175,176], antitumor [177], anti-inflammatory [177,178,179,180], and anticancer effects [29,181,182,183,184] have been reported (Figure 2).

The diverse fucoidan-based carriers have been developed during recent years as nanoparticles for different purposes, such as for the releasing of the antitumor drug curcumin [90,91], the delivery of the anticancer drug doxorubicin [112], the encapsulation of the anticancer drug docetaxel (DTX) [113], for loading anticancer methotrexate (MTX) [114], for the delivery of antimicrobial and anti-inflammatory berberine [185,186,187], for the delivery of antitumor, anti-inflammatory, antioxidant, and hypoglycemic Oncocalyxone A [188], for releasing antibiotic gentamicin [189], for the carrying of basic fibroblast growth factor (bFGF) [190] and stromal cell-derived factor [191] as microparticles for the delivery lipoic acid, as hydrogels for releasing fibroblast growth factor-2 in vitro and in vivo [192], as nanoparticles functionalized with antibody-ErbB-2 for reducing tumor growth of lung [193], as 3D scaffolds for delivery vascular endothelial growth factor [115,116], and as polymeric micelles for loading the antitumor paclitaxel and curcumin [117] (Figure 2).

Exopolysaccharides (EPSs) are marine polysaccharides produced by marine bacteria [194,195,196,197,198], fungi [199,200], and microalgae [201,202,203] with new chemical and physical properties compared to polysaccharides produced by macro algae. The antioxidant function [204,205,206,207,208,209], preventing microbial infections [202,210,211,212,213], anticancer activity [86,92,201,214,215], healing bone [216,217], anticoagulant properties [218], antiviral activity [219,220,221,222,223], anti-inflammatory effects [224], potential for skin or cartilage grafting [222,225], inhibiting osteoclastogenesis [226], and promoting skin wound healing [227] (Figure 2). An overview showed high diversity of biological properties, as well as the high potential of marine polysaccharide as multifunctional carriers in drug delivery systems and tissue engineering. It seems that the future exploration on new structures of marine polysaccharides, as well as their long-term toxicity assays, will prompt the developments of these biomaterials in drug delivery approaches and tissue engineering in near future.

## 5. Food and Feed

### 5.1. Polysaccharides in Food Field

**Figure 3:**
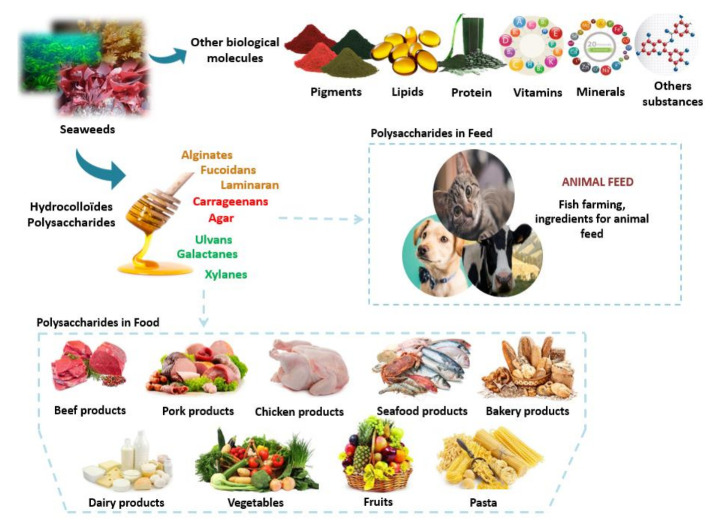
Algal polysaccharides in food and feed industry.

Food hydrocolloids are generally employed for their physical functions in stabilizing emulsions, viscous behavior, gelation, suspensions and foams, and control of crystal growth (Figure 3). The viscosity depends considerably on the preparation method. High temperature is particularly adverse, and the pH needs to be between 6 and 7 [228]. Seaweeds provide numerous several hydrocolloids to the food and feed industries [229], and the most important are agar, carrageenan (from red seaweeds), alginates, and sometimes sulfated fucoidans and laminarins (from brown seaweeds). Depending on their intrinsic structural characteristics and extrinsic environmental factors, polysaccharides frequently exhibit versatile rheological and physicochemical properties, which further affect their applications in food products. Typically, carbohydrates (especially polysaccharides) which present in liquid and/or solid food systems determine their structures and then their functions in industry.

The influence of these biologically active macromolecules on smaller molecules, such as tastant compounds and aroma, has been investigated, with various studies concluding that apparent viscosity changes [230] and the physical entrapment of compounds [231] together explain perceptual differences [232]. These investigations tend to concentrate on the matrix structure and the release characteristics when envisaging changes in food perception.

Several studies have evaluated the effect of the incorporation of algal polysaccharides on nutritional, textural, and organoleptic properties of meat products (e.g., pork, beef, and fish products) (Figure 3). Recently, the antioxidative potential of laminarin (L), fucoidan (F), and an L/F extract from the brown seaweed *Laminaria digitata* was evaluated in pork homogenates and in horse heart oxymyoglobin. The results of this study demonstrated the feasibility of using these two polysaccharides (especially fucoidan) to increase the antioxidant activity of functional cooked meat products and improving the human antioxidant defense systems [233].

In their study, Jensen et al. [234] reported that alginate (used alone or in combination with other hydrocolloids) has an appetite regulator potential and thus could be used as a food supplement. However, according to [235], its incorporation into breakfast bars does not show significant differences as an appetite suppressant, compared to the control.

Albert et al. [236] demonstrated that alginate was also found to be effective as a coating film in microwave-cooked chicken nuggets, improving the heat distribution and thus shortening the cooking time. Sodium alginate was also used as a coating agent of bream, showing great results with additional antioxidant ability, which could delay the decay of the fish and enhance its shelf-life [237]. Coating foods with alginate improved the sensory quality and reduced the loss of water. It has also been reported that adding alginate to melon acts as a carrier for antimicrobials, which improved its shelf-life [238]. Alginates were also applied as a carrier for the anti-browning agents such as ascorbic acid and citric acid, which preserved the color of fresh cut Kent mangoes and improved the antioxidant potential. It has also been reported (i) that coating foods with alginate improved the sensory quality and reduced the loss of water and (ii) that adding alginate to melon acts as a carrier for antimicrobials, which improved its shelf-life [238].

Due to its thickening and gelling properties, its high melting temperatures, as well as its ability to hold into sugar to prevent crystallization, the agar extracted from rhodophyceae is highly sought in the food industry to prepare icings and bakery glazes [239]. The low gel strength matrix formed by agar is a property that makes its use possible in a wide range of food applications, including in liquid and spreadable foods (e.g., soft-texture confectionery) [240], as fat replacers, as cryoprotectants that minimize the damages occurring during the freezing/thawing process [241], and as edible films [232].

Carrageenan, isolated from red seaweeds, can function as a bulking agent, emulsifier, carrier, glazing agent, gelling agent, stabilizer, humectant, or thickener [232]. These sulfated polysaccharides are added to processed foods because it can bind water, promote gel formation, thicken, stabilize, and improve palatability and appearance through interaction with other substances in the food (e.g., carboxylmethyl-cellulose (CMC), galactomannan, starch, sodium, or calcium phosphates and proteins) [241]. Due to their important physicochemical and rheological properties, native carrageenans and semi-refined carrageenans are usually used in the food industry as an ingredient in dairy products such as ice cream, cheese, yoghurt, and milk-based products [242,243,244,245].

Other works have demonstrated the use of carrageenans in bakery products such as bread [246] and as a coating film to extend the shelf-life of fresh chicken breast [247].

Moreover, Piculell [241] showed that carrageenan can prevent separation and maintain texture in dairy products when added in small amounts of around 0.3% in milk gels (such as creamy fillings, flans, and custards), yoghourt, whipped cream, and milkshakes, and around 0.03% in liquid milk products and frozen desserts.

This sulfated polysaccharide can be used as a fat substitute in processed meats, as it restores tenderness and improves moisture retention in low-fat processed meats such as hamburgers [248]. For example, in their research, Kumar and Sharma [249] showed that ground pork patties with less than 10.0% (*w*/*w*) total fat and carrageenan at important concentrations of 0.75% (*w*/*w*) actually had higher moisture retention after cooking and a similar texture compared to pork patties containing 20.0% (*w*/*w*) fat without carrageenan. It has been shown that carrageenan is successful in controlling discoloration, maintaining texture through shelf-life, and providing antibacterial protection when used as an edible fruit coating on sliced lychee bananas and mangoes [250].

### 5.2. Polysaccharides in Feed Field

Bioactive polysaccharides and oligosaccharides (BPO) are classified as a kind of indigestible but fermentable natural macromolecular carbohydrate. BPO are characterized by being biocompatible and biodegradable, along with their antibacterial, antioxidant, immunostimulating, and metabolic regulatory activities [1]. Because of these features, they can be used as effective alternatives to antibiotics in modulating gut microbiome. Several studies have investigated the prebiotic effects of oligosaccharides and polysaccharides derived from seaweeds in rats or mice being fed a seaweed-supplemented diet. Results conducted by Liu et al. [251] demonstrated a raise in the abundance of beneficial gut microbes such as *Bifidobacterium breve* and a diminution in pathogenic bacteria such as *Clostridium septicum* and *Streptococcus pneumonia* in rats supplemented with water-soluble polysaccharides from the red seaweed *Chondrus crispus*. Moreover, an increase in short chain fatty acids (SCFA) production and colonic growth was obtained, as well as an improvement of host immunity modulation through an elevation of the plasma immunoglobulin levels.

The supplementation of diets with extracts of the brown seaweeds *Undaria pinnatifida* and *Laminaria japonica* has resulted in suppressed weight gain of rats, influenced by the composition of gut microbial communities associated with obesity by a reduction in the ratio of Firmicutes to Bacteroidetes and reduced populations of pathogenic bacteria, including *Clostridium*, *Escherichia* and *Enterobacter genera* [252]. From Lean et al. [253], the oral administration of fucoidan from brown seaweeds has been shown to reduce the inflammatory pathology associated with dextran sulfate sodium (DSS)-induced colitis in mice, indicating its important potential for treating inflammatory bowel disease. Furthermore, Kuda et al. [254] showed that rats fed with a diet containing laminarin and low M_W_ alginate isolated from pheophyceae suppressed the production of indole, p-cresol, and sulfide, which are the putative risk markers for colon cancer. The neoagaro-oligosaccharides derived from the hydrolysis of agarose by β-agarase enzyme resulted in a rise in the numbers of *Lactobacillus* and *Bifidobacterium* in the feces or cecal content of mice, along with a decrease in putrefactive bacteria [255].

## 6. Antimicrobial/Antiviral Agents

The biodegradability, biocompatibility, and non-toxic nature of polysaccharides isolated from natural sources, make them valuable ingredients in different fields, such as pharmaceuticals, nutraceuticals, food, or cosmetic industries. They have been used in healthcare, namely in cancer diagnosis and treatment, in drug delivery, in tissue engineering, and as antimicrobial and antiviral agents [256].

In this section, a compilation of the literature of polysaccharides from natural sources with antimicrobial/antiviral potential were considered, as described in Table 3.

The great potential of polysaccharides from different sources as antiviral agents is undeniable, especially when the origin is seaweeds. Alginate, fucoidan, and laminarin, typically obtained from brown algae [95,256,257,258,259,262,271,275,287], carrageenan and galactans from red algae [257,258,261,262,263,264,265], and ulvan from green algae [262,266], often present antiviral activity against several viruses (Table 3). Indeed, this recognized biological activity immediately aroused the interest of the scientific community to search for solutions against the new SARS-CoV-2 virus responsible for causing COVID-19 [264,271,288]. Several polysaccharides have been explored with this propose, as is the case for carrageenan and fucoidan, from algae and heparin, with animal origin, that have already shown promising results against this virus [257,262,271,281]. Different antiviral mechanisms are associated to these compounds, such as the inhibition of enveloped and nonenveloped viruses through the inhibition of the binding or internalization of the virus into the host cells, the inhibition of virus replication through the suppression of the DNA polymerase activity, among others [257]. The role of heparin, however, should be highlighted. This polysaccharide, usually used as anticoagulant agent, is also known to contribute as an antiviral agent. For SARS-CoV-2, studies have shown that this bioactive compound strongly binds to the Spike protein, avoiding the entry in the host cells. Particularly for viruses causing respiratory problems, heparin also prevents pulmonary thrombosis, suggesting that heparin may act through multiple mechanisms [281].

Regarding the potential of polysaccharides against microbes other than viruses, the antibacterial activity stands out when compared with antifungal activity (Table 3). Several polysaccharides were found to be effective against a wide range of pathogenic bacteria. Between them, almond gum [260], carrageenan [263,265], laminarin [259], pectin [256], and polysaccharides obtained from species of *Ganoderma* sp. [285] demonstrated the ability to inhibit the growth of species as *Listeria monocytogenes*, *E. coli*, *Pseudomonas aeruginosa*, *Klebsiella pneumonia*, *Chlamydia trachomatis*, *Staphylococcus aureus*, *Salmonella typhimurium*, *Helicobacter pylori*, *Prevotella. intermedia*, *Porphyromonas gingivalis*, among many others known to cause infections in humans.

A similar scenario is not observed for the antifungal activity of polysaccharides, possibly not due to the lack of activity, but due to the lower number of studies including this type of organisms. However, fucoidan and ginseng showed not only antiviral and antibacterial activity, but also antifungal activity as well [276,277,278]. Together with *Ganoderma* polysaccharides [285], pathogens extremely relevant not only for human health (as *Candida albicans*, *Aspergillus fumigatus*, and *A. flavus*), but also for agriculture (as *A. niger* and *Penicillium digitatum*), were identified, making them great candidates for the pharmaceutical and phytopharmaceutical industries. Taking this information in consideration and knowing that fungi are organisms capable of inflicting significant losses in several fields, more research should be considered in an attempt to find compounds able to control them.

In addition to the antimicrobial/antiviral potential of the polysaccharides presented in Table 3, other polysaccharides are known to indirectly contribute to those activities, as drug delivery carriers or even by stimulating defense mechanisms of hosts against pathogens or the growth of the regular microbiota; this fact is also extremely relevant for the pharmaceutical industry as well. The seaweed polysaccharide laminarin is known to stimulate the defense mechanisms of plants that are involved in the cascade of genes encoding proteins related with antimicrobial properties [289]. In the case of pullulan, a polysaccharide produced by the fungus *Aureobasidium pullulans*, its chemical structure is easily modified to deliver different drugs in the form of microparticles, nanoparticles, and hydrogels, among others [290]. Similarly, polysaccharides such as agar, hyaluronan, konjac glucomannan, schizophyllan, bacterial cellulose, xanthan gum, among others, have also been explored with this objective, presenting different therapeutic targets [279,291,292]. Another case is inulin, a polysaccharide produced by plants that is responsible for enhancing the proliferation of bacteria such as Bifidobacteria, known to be health-promoting, suppressing the growth of potential pathogens in the gut, and also acting as immunomodulators [293]. Although antibacterial activity is not described for levan, it has been proposed as a potential compound for the treatment of peptic ulcers typically associated to bacteria such as *Helicobacter pylori*. Due to the high adhesion ability and prebiotic activity, it has the capacity to protect and prevent the development of peptic ulcers [294].

Another feature to be explored in this field is the possibility to create synergisms between different compounds. A study conducted with fucoidan and antibiotics against oral pathogenic bacteria showed that the combination of this polysaccharide with antibiotics lead to an increase in the rate of the elimination of colony forming units per milliliter, when compared with the results obtained for the antibiotics or polysaccharides alone [275].

All these possibilities show the great potential of polysaccharides from different natural sources as antimicrobial and antiviral agents, not only in a direct way but also through different mechanisms that contribute to the same goal.

## 7. Chemical, Chemo-Enzymatic, and Enzymatic Functionalization of Polysaccharides

Polysaccharides can have various biological activities, for example, antiviral, antitumor, antioxidant, and immunomodulatory [4,9,13], which depends on their structure [4,36,295]. Polysaccharides have many highly reactive groups (acetamido, amino, carboxyl, hydroxyl groups) [296], which can be used in various functionalization ways. The biological activity of polysaccharides can be significantly increased with properly selected methods and conditions for carrying out structural modification [297,298].

### 7.1. Chemical Functionalization

Chemical modification, in general, is the introduction of various functional groups into the polysaccharide structure [295,296,297,298,299]. This often leads to a decrease in the molecular weight of the polymer [300] and, consequently, an increase in its solubility in water [299]. The biological properties of modified polysaccharides depend not only on the method of chemical modification but also on the degree of substitution. Moderate substitution can enhance the biological activity of the polysaccharide [300]. In this case, a concentration-dependent effect of the activity of the carbohydrate polymer was observed [300,301].

One of the most common methods for modifying polysaccharides is sulfation. Polysaccharides functionalized in this way have higher immunoregulatory, antiviral, anticoagulant, antitumor, and antioxidant activities [298,302].

Another method of moderate substitution is the acetylation of polysaccharides. Acetyl groups can cause polysaccharide branches to stretch and change orientation, causing the formation of polysaccharide molecules with a transverse order [299]. As a result of a spatial arrangement change in the polysaccharide chains, the properties of macromolecules also change. It was found that the introduction of acetyl groups significantly increased the antioxidant [300,301], immunomodulatory [300,303,304], and anti-inflammatory [303] activities of the polysaccharides.

Phosphorylation and benzoylation can also increase the antioxidant activity of polysaccharides [305,306,307]. Xu et al. [15] demonstrated that acetylated and benzoylated polysaccharides were more effective in lowering blood glucose levels in mice when used as a cardioprotective agent compared to the native polymer.

Carboxymethylation can increase the antioxidant, antitumor, immunoregulatory, and antibacterial functions of polysaccharides [308]. It was shown in [309] that carboxymethylation converted polysaccharides into water-soluble products. Chen et al. [300] showed that carboxymethylation could slightly increase the ability to inhibit the discoloration of β-carotene. There was also a slight increase in the effect on the pinocytic activity of peritoneal macrophages in mice, which decreased the effect of the TNF-α protein on the secretion.

If necessary, it is possible to select the modification conditions with no decrease in the molecular weight of the polymer. The authors of [310] described a method for dextran phosphorylation, which did not lead to polysaccharide chain degradation. Silva et al. [311] determined the optimal conditions for carboxymethylation in which the least chain degradation was observed.

Chemical functionalization is the most common among the numerous methods for modifying polysaccharides due to the vast possibilities of introducing various functional groups into the structure of the polymer molecule. However, along with the advantages, this type of modification has some disadvantages. The major one is the frequent toxicity of the chemicals used.

### 7.2. Chemo-Enzymatic Functionalization

Enzymes can be used as catalysts for the attachment of functional groups employing chemical reagents, and can themselves act as a functionalizing component, connecting with a polysaccharide molecule using a cross-linking agent [312]. Compared with the method of chemical binding, the method of modification catalyzed by enzymes is cheaper and safer [313].

The polysaccharides were modified with phenolic acids (gallic [314], caffeic [314,315], and ferulic [316]) in the presence of laccase as a catalyst. Chitosan molecules functionalized with phenolic acids showed much higher antioxidant activity in scavenging the radical cations 2,2′-Azino-bis-(3-ethylbenzothiazoline-6-sulfonic acid) (ABTS) [314] and 2,2-diphenyl-1-picrylhydrazyl (DPPH) [315 and, under certain modification conditions, a higher antimicrobial activity against *E. Coli*, as well as antifungal activity against *C. albicans* [314].

Lipase is often used to acylate the hydroxyl groups of polysaccharides [317,318,319]. Lipase, possessing regioselectivity, gives direction to the process of the structural modification of the polysaccharide [319].

Peroxidases are used to catalyze oxidative polymerization and graft the functional molecules to polymers in a two-step reaction. The first step involves the generation of radicals by peroxidase, then reactively oxidized target molecules crosslink the polymer [320]. Li et al. [321] reported that lytic polysaccharide monooxygenases could efficiently supply H_2_O_2_ in situ to peroxidases using a gallic acid substrate for the functionalization of chitosan. In [322], a pectin polysaccharide was enzymatically modified by crosslinking ferulic acid groups using horseradish peroxidase. The resulting polysaccharides showed an improved ability to stabilize oil-in-water emulsions against coalescence and flocculation [322].

Tegl [315] demonstrated a chemo-enzymatic functionalization with glucose oxidase as a modifying reagent of particles of chitosan–zeolite and chitosan–zeolite modified with caffeic acid. Chitosan–zeolite particles modified with caffeic acid and then glucose oxidase demonstrated enhanced combined antioxidant and antimicrobial activity compared to the polysaccharide without modifications.

### 7.3. Enzymatic Functionalization

Enzymatic processing avoids the use of substances that are aggressive and harmful to human health. Enzymes are suitable tools for changing the structure of polysaccharides due to their specificity. Enzymatic technologies have great potential for modifying the properties of natural food ingredients such as polysaccharides to improve their functional characteristics [318]. However, the possibilities of such a modification are very limited and often come down to the cleavage of various constituents of the polysaccharide chain, the shortening of the chain, and, as a consequence, to a decrease in the values of molecular weight and intrinsic viscosity.

Oosterveld et al. [323] showed enzymatic deacetylation of pectin polysaccharide using pectin methylesterase, pectin acetylesterase, rhamnogalacturonan acetylesterase, arabinofuranosidase B, and rhamnogalacturonase in various combinations. The modification of rhamnogalacturonans and arabinans with enzyme mixtures such as endo-arabinase plus arabinofuranosidase, rhamnogalacturonase plus rhamnogalacturonan acetylesterase, and polygalacturonase plus pectin methyl esterase resulted in a decrease in molecular weights and intrinsic viscosity [323]. It was shown in [324] that the use of pronase E to modify exopolysaccharides isolated from lactic acid bacteria did not affect the molecular weight of the studied samples and, therefore, was suitable for protein removal.

Thus, numerous methods for modifying polysaccharides lead to a wide range of polymers with different biological properties. When modifying polysaccharides, it is possible to weaken their biological properties [300,314,316]. For this reason, the modification conditions and methods should be selected depending on the functionalization goals. It is essential to select the optimal conditions for the modification to obtain polymers with improved biological properties.

## 8. Current Markets

Long and laborious processes associated with both technology-transfer and regulatory constraints in taking a substance from research to the market is one of the main explanations for the fact that the list of carbohydrates with biotechnologically relevant properties currently on the market is shorter than that of all carbohydrates being researched for their biological activities. A compilation of currently marketed bioactive polysaccharides was performed and presented in Table 4.

Noteworthy, most carbohydrate polymers in the market serve at least one of three main functions as a physico-chemical formulae modifier (e.g., thickener, stabilizer, binder, or emulsifier in food, feed, or cosmetic products), as a nutraceutical supplement with health claims, or as a pharmaceutical product. Importantly, more often in the case of nutraceuticals but also sometimes in the case of pharmaceuticals, some of the health claims are supported in preliminary scientific research (e.g., in vitro models) or even in the absence of scientific evidence (e.g., cultural or traditional medicine beliefs).

Many of the polymers detailed in Table 4 are sold in their purified form (e.g., heparin, pectin, carrageenan, among others), while some of the noted polysaccharides are not explicitly sold as such (e.g., *Astragalus* polysaccharide). In the case of the latter, it is often found that the whole source organism (e.g., in powder) or an extract of the source organism (standardized for a certain percentage of polysaccharides) is sold instead. In such cases, it becomes harder to quantify and characterize the market. An example of this difficulty is that of ginseng’s polysaccharides. Despite being recognized in academia as one of the main bioactive components in ginseng products [325], ginseng itself as a whole product or in the form of extracts (which contain many more metabolites than the polysaccharides alone) are so popular that not one single polysaccharide-specific product could be found—it is for this reason that it is not included in Table 4.

Virtually all the listed polysaccharides in Table 4 present different variations in properties/claims and applications according to the specific source organism and method of extraction/refinement. Due to these two variables, polymers with different molecular weights and degrees of ramification are obtained, which in turn present different bioactivities.

Due to an increased demand from consumers and rapid innovation in the fields of food, feed, cosmetics, and biopharmaceuticals technologies, as with most natural products, the markets for the listed polysaccharides are growing. This growth, however, has been modified (either positively or negatively) by the recent events of the COVID-19 pandemic. Low molecular weight heparin has been used prophylactically and therapeutically in COVID-19 patients [326], which has contributed to an increase in demand for this product (https://www.alliedmarketresearch.com/heparin-market-A06186 (accessed on 4 September 2021). On the other hand, non-essential polysaccharide products directly related to businesses that were shut down during the pandemic (e.g., xanthan gum in the beauty industry) observed a decrease in growth rate due to COVID-19 (https://www.theinsightpartners.com/reports/xanthan-gum-market (accessed on 4 September 2021).

The group of animal-based polysaccharides is, by far, the largest market for these polymers, with a combined market value superior to 20 billion USD in 2020 (https://www.alliedmarketresearch.com/heparin-market-A06186 (accessed on 4 September 2021), https://www.reportsanddata.com/report-detail/hyaluronic-acid-market (accessed on 4 September 2021), https://www.bccresearch.com/market-research/plastics/chitin-chitosan-derivatives-markets-report.html. (accessed on 4 September 2021), https://www.grandviewresearch.com/industry-analysis/chondroitin-sulfate-market (accessed on 4 September 2021). Of these, hyaluronic acid is the polymer with the largest market size (9.6 billion USD in 2020) (https://www.reportsanddata.com/report-detail/hyaluronic-acid-market (accessed on 4 September 2021), followed by heparin (6.5 billion USD in 2020) (https://www.alliedmarketresearch.com/heparin-market-A06186 (accessed on 4 September 2021), chitin/chitosan (projected 4.2 billion USD in 2021) (https://www.bccresearch.com/market-research/plastics/chitin-chitosan-derivatives-markets-report.html), and chondroitin sulfate (1.2 billion USD in 2020) (https://www.grandviewresearch.com/industry-analysis/chondroitin-sulfate-market (accessed on 4 September 2021). The fact that animal-based polysaccharides are of such value is directly related to their value per kilogram, which is high due to their medical-grade processing requirements, along with the high-volume of sales of chitin/chitosan in the field of water treatment. Plant-based gums/fibers, as well as seaweed phycocolloids, have much higher volumes of sales, but lower prices per kilogram, given the less refined nature of these mostly food-grade polymers. An example of such a polymer is agar, which, despite its high volume of sales in kilograms, has a market size estimated of 239 million USD (in 2020) (https://www.industryarc.com/Research/Global-Agar-Market-Research-509553 (accessed on 4 September 2021). Nonetheless, the plant-based polysaccharide inulin, given its use as a nutraceutical prebiotic fiber, has a higher price range, rendering a market of 2.35 billion USD in 2020 (https://www.grandviewresearch.com/industry-analysis/inulin-market (accessed on 4 September 2021). The markets for three of the most popular food-grade thickening agents are each under the 1 billion USD threshold, namely pectin (888 million USD in 2020) (https://www.mordorintelligence.com/industry-reports/pectin-market (accessed on 4 September 2021), alginate (728 million USD in 2020) (https://www.grandviewresearch.com/industry-analysis/alginate-market (accessed on 4 September 2021), and carrageenan (742 million USD in 2019) (https://www.grandviewresearch.com/industry-analysis/carrageenan-market (accessed on 4 September 2021). An alternative to these polymers is bacterial xanthan gum, which has an increasing market share, and is currently valued at 576 million USD (in 2020) (https://www.theinsightpartners.com/reports/xanthan-gum-market (accessed on 4 September 2021). Polysaccharides, discovered later and hence newer in the market, have smaller market sizes, such as the bacterial and fungal polysaccharides, e.g., dextran (191 million USD in 2019) (https://www.industryresearch.co/global-dextran-market-18478845 (accessed on 4 September 2021), bacterial cellulose (390 million USD in 2020) (https://www.industryresearch.co/global-microbial-and-bacterial-cellulose-market-18720450 (accessed on 4 September 2021), and pullulan (126 million USD in 2020) (https://www.industryresearch.co/global-pullulan-market-18823725 (accessed on 4 September 2021). Despite presenting high growth rates, those with very low volumes of sales (strictly nutraceuticals, for instance) present much smaller markets, such as seaweed-based fucoidan (projected 30 million USD in 2020) (https://www.360researchreports.com/global-fucoidan-market-14030270 (accessed on 4 September 2021) and laminarin (2 million USD in 2019) (https://www.360researchreports.com/global-laminarin-market-18049714 (accessed on 4 September 2021) or fungi-based lentinan (10 million USD in 2019) (https://www.360marketupdates.com/global-lentinan-market-14829124 (accessed on 4 September 2021) (Table 5).

## 9. Conclusions and Future Perspectives

Polysaccharides have received a great deal of attention during the two last decades notably with the development of glycosciences and glycobiology. To be realistic, despite the large number of bioactive polysaccharides identified and fully or partially characterized, only a small number of them have found significant commercial application. The reasons for this are numerous, but the main ones are probably their costs, their uncertain structures, their polydispersity, the maintenance of their quality, which may be difficult, and the presence of the market of competitive bioactive polysaccharides. For that, except bioactive polysaccharides having highly specific biological activities with no competitor on the market, it is very difficult for new ones to access to a viable business model.

A better understanding and resolving of their structures with modern analytical tools including NMR spectroscopy, mass spectrometry (ESI-IT MS, ESI-Q-TOF MS, MALDI-TOF MS, and others), HPAEC, GPC-MALLS, infrared spectroscopy, etc., opens the way for a better understanding of the relations between structures and biological functions. Accumulating data from these different structural analysis techniques sometimes with hyphenated approaches (LC-MS or LC-NMR) reinforces this opportunity. However, the main drawback that has up to now limited the development of bioactive polysaccharides in several fields of applications, notably the therapeutic one, is their polydispersity, and, for the majority of them, the impossibility to propose a full and monodispersed structure. The obtaining of fully purified characterized oligosaccharides could be a good opportunity to solve this problem. Indeed, the correlation of a structure–activity relationship could help to produce biomimetic polysaccharides using non bioactive polysaccharides after their controlled modification using chemistry, biochemistry, or physics. In this field, enzymatic modifications are very promising as they target specific chemical groups to engineer new polysaccharide with a controlled structure from native polysaccharide extracted from plants, algae, and others. Polysaccharides have also received a great deal of attention as bioactive materials for high value applications benefiting from strong development of additive manufacturing. Another opportunity for bioactive polysaccharides is the recent development of technologies allowing the culturing of some microorganisms as new EPS producers. In this field, the photobioreactors technology and the creation of startups exploiting these microorganisms for the production of original polysaccharides is booming. However, their costs of production currently still limit their commercialization, albeit only in the field of cosmetic.

## Figures and Tables

**Table 1:** Immunomodulatory activity of polysaccharides with different structures.

Polysaccharide Components	Mw (kDa)	Immunomodulatory Activity	Sources
residues of arabinose, glucose, galactose, low content of protein components	401	did not have cytotoxicity, increased NO production, promoted the proliferation of spleen lymphocytes	[18]
residues of arabinose, glucose, galactose, low content of protein components	99	did not have cytotoxicity, increased NO production, promoted the proliferation of spleen lymphocytes	[18]
residues of uronic acid, arabinose, galactose, and glucose, low content of protein components	99	did not have cytotoxicity, increased NO production, promoted proliferation of spleen lymphocytes and proliferation of T-lymphocytes	[18]
residues of uronic acid, galactose, arabinose, and glucose, low content of protein components	43	did not have cytotoxicity, increased NO production, promoted proliferation of spleen lymphocytes and proliferation of B-lymphocytes	[18]
residues of glucose, mannose, ribose, galactose, xylose, and arabinose	-	increased survival of L02 cells caused by H_2_O_2_	[22]
residues of fucose, galactose, and 3-O-methylgalactose	120	increased NO production, stimulated splenocytes	[25]
type II arabinogalactan, galacturonic acid residues	-	did not have cytotoxicity, increased NO production by J774. A1 macrophage cells, and increased cytokine production	[34]
residues of d-mannose, d-glucose	394	had a stimulating effect on macrophage cells RAW 264.7, at high concentrations decreased cell viability, increased NO production, stimulated splenocytes and T-lymphocytes, had a protective effect against macrophage apoptosis caused by H_2_O_2_	[43]
residues of d-mannose, d-glucose	362	had a stimulating effect on macrophage cells RAW 264.7, at high concentrations decreased cell viability, increased NO production, stimulated splenocytes and T-lymphocytes, had a protective effect against macrophage apoptosis caused by H_2_O_2_	[43]
galacturonic acid, arabinose, and galactose	-	increased the production of cytokines IL-1α and G-CSF	[46]

**Table 2:** Alginate or oligoalginates generate resistance in different plants against diverse biotic and abiotic stresses.

Plant	Polysaccharide	Dose	Application Mode	Effect	Metabolism	References
**Wheat**	Alginate oligosaccharides	1000 mgL^−1^	Supplementation in the growth medium	Tolerance to drought stress	-Enhancement of antioxidant system -Activation of related genes involved in ABA signal pathway (LEA1, SnRK2 and P5CS)	[67]
**Kiwi fruit**	Alginate oligosaccharides	50 mgL^−1^	Fruit soaking	Disease resistance to gray mold caused by *Botrytis* *cinerea*	-Enhancement of antioxidant system -Inducing of defense-related enzymes activities (PPO, PAL, and GLU)	[68]
**Rice**	Alginate oligosaccharides	1 mgmL^−1^	Foliar spraying	Disease resistance to *Magnaporthe grisea*	-Activation of PAL, POD and CAT activities	[69]
**Safflowr**	Alginate	0.075% and 0.15% (w/v)	Supplementation in growth medium	In vitro tolerance to salt stress	-Production of secondary metabolites (TPC, TFL, TFD, and Ant) -Enhancement in the antioxidant activity (CAT, TAC, and PAL)	[70]
***Arabidopsis thaliana***	Alginate Oligosaccharide	25 mgL^−1^	Foliar spraying	Resistance to Pst DC3000	-Production of early signal molecules (ROS, NO) - Activation of SA pathway	[71]
**Cucumber**	Alginate oligosaccharides	0.2% (w/v)	Foliar spraying	Water stress tolerance	-Decrease on MDA and (•OH) content -Activation of SOD and POD activities -Activation of genes involved in ABA signaling pathway	[72]

LEA1: Late embryogenesis abundant protein 1 gene, SnRK2: Sucrose nonfermenting 1-related protein kinase 2 gene, P5CS: Pyrroline-5-Carboxylate Synthetase gene, PPO: polyphenoloxidase, PAL: Phenylalanine ammonia lyase, GLU: β-1,3-glucanase, POD: Peroxidase, TPC: Total phenolic content, TFL: Total flavonoids (TFD), TFL: Total flavonols, Ant: Anthocyanin, TAC: Total antioxidant capacity, ROS: Reactive oxygen species, NO: Nitric oxide, SA: Salicylic acid, ABA: Abscisic aid.

**Table 3:** Main polysaccharides from natural sources with antimicrobial and antiviral potential.

Polysaccharides	Source		Biological Activity	Species	References
Alginate/alginic acid	Seaweed		Antiviral	HIV-1, HPV, DENV, HSV	[257,258,259]
Almond gum	Plant		Antibacterial	*Bacillus thuringiensis, Klebsiella pneumonia, Bacillus subtilis, Pseudomonas aeruginosa, Listeria monocytogenes*	[260]
Carrageenan	Seaweed		Antibacterial	*Chlamydia trachomatis, Saccharomyces cerevisiae, Staphylococcus aureus, Bacillus cereus, Escherichia coli, Pseudomonas aeruginosa*	[257,258,261,262,263,264,265]
			Antiviral	HPV, HSV-1, HSV-2, HIV, HRV, Influenza A, DENV-2, DENV-3, VZV, RABV, EV71, SARS-CoV-2	
Ulvan	Seaweed		Antibacterial	*Enterobacter cloace, Escherichia coli*	[262,266]
			Antiviral	NDV, JEV	
Rhodophyta Galactans	Seaweed		Antiviral	HSV-1 and HSV-2, DENV-2, HIV-1 and HIV-2, HAV, HPV, DENV	[257,259,267]
Calcium spirulan	Cyanobacteria		Antiviral	HSV-1, HCMV, Influenza A, Coxsackie virus, MV, HIV-1, PV, Mumps virus, HPV, DENV	[257,259,268]
Nostoflan	Cyanobacteria		Antiviral	HSV-1, HSV-2, HCMV, Influenza A	[257,259,269,270,271]
Chitin/chitosan	Animal		Antibacterial	*Escherichia coli, Vibrio cholerae, Shigella dysenteriae, Bacteroides fragilis*	[272,273]
Dextran	Bacteria		Antiviral	HPV	[258,266,274]
			Antifungal	*Candida albicans*	
Fucoidan	Seaweed		Antibacterial	*Listeria monocytogenes, Micrococcus luteus, Staphylococcus aureus, Salmonella typhimurium, Streptococcus mutans, Streptococcus sanguinis, Streptococcus* *sobrinus, Strongyloides ratti, Streptococcus criceti, Streptococcus anginosus, Streptococcus gordonii, Aggregatibacter actinomycetemcomitans, Fusobacterium nucleatum,* *Prevotella intermedia, Porphyromonas gingivalis*	[95,257,262,267,271,275,276]
			Antiviral	HIV, HSV-1, HSV-2, DENV, *HCMV*, NDV, SARS-CoV-2	
			Antifungal	*Aspergillus flavus, Aspergillus fumigatus, Mucor sp.*	
Ginseng’s polysaccharide	Plant		Antibacterial	*Helicobacter pylori, Bacillus cereus, Staphylococcus aureus, Pseudomonas aeruginosa, Listeria monocytogenes, Salmonella enteritidis, Escherichia coli, Streptococcus pneumoniae*	[277,278]
			Antifungal	*Candida albicans*	
			Antiviral	H1N1 Influenza virus, H5N1 Influenza vírus, HIV, HBV, RSV	
Heparin	Animal		Antiviral	HPV, SARS-CoV-2	[258,279,280,281]
Laminarin	Seaweed		Antibacterial	*Escherichia coli, Listeria monocytogenes, Staphylococcus aureus, Salmonella typhimurium*	[257,259]
			Antiviral	HBV, HIV-1	
Lentinan	Fungi		Antiviral	SARS-CoV-2	[282]
Levan		Bacteria	Antiviral	(HPAI) A(H5N1), ad40	[283]
Pectin	Plant		Antibacterial	*Citrobacter* sp., *Salmonella* sp., *Enterobacter* sp., *Shigella* sp., *Proteus* sp., *Klebsiella* sp.	[256]
*Polygonum multiflorum*’s polysaccharide	Plant		Antiviral	Coronavirus	[284]
*Ganoderma* polysaccharides	Fungi		Antibacterial	*Erwinia carotovora, Bacillus cereus, Acinetobacter aerogenes, Acrobacter aerogenes, Arthrobacter citreus, Bacillus brevis, Bacillus subtilis, Corynebacterium insidiosum, Escherichia coli, Proteus vulgaris, Clostridium pasteurianum, Micrococcus roseus, Mycobacterium phlei, Staphylococcus aureus*	[285]
			Antifungal	*Penicillium digitatum, Aspergillus niger*	
Xylan	Plant		Antibacterial	*Klebsiella pneumoniae*	[286]
			Antiviral	HSV	

*HIV*—Human immunodeficiency virus, *HPV*—Human papillomavirus, *DENV*—Dengue virus, *HSV*—Herpes simplex virus, *HRV*—Human Rhinovirus, *VZV*—Varicella zoster vírus, *RABV*—Rabies virus, *EV*—Enterovirus, *SARS-CoV-2*—Severe Acute Respiratory Syndrome Coronavirus-2, *NDV*—Newcastle disease vírus, *JEV*—Japanese encephalitis virus, *HAV*—Hepatitis A, *HCMV*—Human cytomegalovirus, *MV*—measles virus, *PV*—Poliovirus, *HBV*—Hepatitis B virus, *RSV*—respiratory syncytial virus, *(HPAI) A(H5N1)*—Highly Pathogenic Asian Avian Influenza A(H5N1) Virus, *ad40*—Adenovirus type 40.

**Table 4:** Currently marketed bioactive polysaccharides along with their biological origin and the main properties for which they are sold.

Polysaccharide	Mainly Sold As/For
**Animal Origin**	
Heparin	Anticoagulant (medical practice) Scientific research (several novel medical uses)
Chondroitin sulfate	Treatment of osteoarthritis in humans and other animals Treatment of cataracts Nutraceutical for cartilage, joint, and bone health ^1^
Hyaluronic acid ^2^	Treatment of osteoarthritis and cataracts Topical pharmaceutics (e.g., dry skin, dry eyes, wounds, or oral inflammation) Plastic surgery filler Cosmetic formulations (anti-aging properties)
Chitin/chitosan ^3^	Sizing and strengthening paper Plant priming Soil fertilizer/conditioner Wine fining Water treatment Biomaterial (e.g., food packaging or wound dressings) Nutraceutical for weight loss ^1^ Nutraceutical with cholesterol lowering effect Scientific research (several novel uses)
**Plant Origin**	
Pectin	Gelling agent Stabilizer Dietary fiber (supplement) with cholesterol lowering effect Dietary fiber (supplement) that delays gastric emptying ^1^ Dietary fiber (supplement) that ameliorates constipation ^1^
Konjac glucomannan	Thickening agent Emulsifier Dietary fiber (supplement) with cholesterol lowering effect Dietary fiber (supplement) that ameliorates constipation Nutraceutical for weight loss ^1^
*Astragalus* polysaccharide	Nutraceutical for immune system stimulation Nutraceutical with diuretic effect Nutraceutical with antiviral activity Nutraceutical with several other medical applications ^1^
Xylan	Source for xylitol production Plastic additive Scientific research (novel packaging solutions and biomedical applications) Cosmetic ingredient
Inulin	Nutraceutical with prebiotic and gut health promoting activitiesNutraceutical with antidiabetic activity Nutraceutical with weight loss activity Dietary fiber (supplement) that ameliorates constipation Food ingredient to increase fiber content and substitute sugar, starch, and fats
*Polygonum multiflorum* polysaccharide	Nutraceutical for hair strengthening and color restoration ^1^ Nutraceutical for neuroprotection ^1^ Nutraceutical with several other medical applications ^1^
Guar gum	Nutraceutical to increase fiber intake (also, indirect method of weight loss) Nutraceutical with cholesterol lowering effect Food ingredient to thicken without gluten, gelatin, or eggs Dietary fiber (supplement) that ameliorates constipation or diarrhea Multiple uses in paper and textile industries Thickener (multiple industries) Stabilizer (food industry) Binder (multiple industries) Laxative
**Seaweed Origin**	
Alginate/Alginic acid	Superabsorbent Plant priming Drug-release carrier Inoculant carrier Thickening agent (multiple industries) Stabilizer (multiple industries) Gelling agent (multiple industries) Emulsifier (multiple industries) Biomedical scaffolding Anti-poisoning ^1^ Nutraceutical with cholesterol lowering effect ^1^ Nutraceutical with anti-hypertensive effect ^1^ Nutraceutical to increase fiber intake (also, indirect method of weight loss) ^1^ Ingredient in peel-off skin masks Reflux treatment
Carrageenan	Gelling agent (multiple industries) Thickening agent (multiple industries) Stabilizer (multiple industries) Emulsifier (multiple industries) Lubricant Inactive excipient in pharmaceutical formulae Immobilization agent for cells/enzymes (biotechnology) Pro-inflammatory agent (scientific research)
Agar	Gelling agent (multiple industries) Food ingredient to solidify liquids Dietary fiber (supplement) that ameliorates constipation or diarrhea Scientific research (molecular biology and microbial/plant culture media)
Fucoidan	Nutraceutical with immunostimulant activity Nutraceutical with anticancer activity ^1^ Nutraceutical with anti-inflammatory activity ^1^ Nutraceutical with antihypertension and antihypercholesterolemia activity ^1^ Nutraceutical with anticoagulant and antithrombotic activities Nutraceutical with antioxidant activity
Laminarin	Reagent for scientific research (bioactivities and enzyme activity) Cosmeceutical with moisturizing capacity Dietary fiber (supplement)Nutraceutical with some health-related claims ^1^
*Caulerpa* sulfated polysaccharide	Nutraceutical with immunostimulation activity Nutraceutical with anticoagulant activity
**Bacterial Origin**	
Dextran	Pharmaceutical for hypovolaemia treatment Antithrombotic and blood thinner Medical lubricant Pharmaceutical for parenteral nutrition/blood substituent Research (several laboratory applications)
Levan	Cosmeceutical ingredient with haircare and skin-whitening properties Food ingredient (dietary fiber supplement and sweetener) Scientific research (bioactivities, e.g., prebiotic, anti-inflammatory, and antimicrobial)
Curdlan	Gelling agent (multiple industries) Water-holding and stabilizing agent (food industry) Scientific research (several therapeutic potentials)
Bacterial cellulose	Thickener (multiple industries) Stabilizer (multiple industries) Modern wound dressings Several medical applications as structural material
Xanthan Gum	Thickener (multiple industries) Stabilizer (multiple industries) Scientific research (biomedical scaffolding)
Gellan gum	Thickener (multiple industries) Emulsifier and stabilizer Drug-release carrier and cell encapsulation agent Agar-substitute in culture media
**Fungal Origin**	
Pullulan	Edible film-forming polysaccharide (oxygen-barrier) Low-calories, tasteless food ingredient (bulking fiber, antifungal) Adhesive and binder (multiple industries) Drug-release agent
Scleroglucan	Thickening agent (food industry) Suspension agent (food industry) Water-holding agent (cosmeceutical industry) Immunostimulant (especially against fungal infections) Edible films Binder in tablets and drug-release carrier Nutraceutical with hypolipidemic and hypoglycemic activities Artificial tears and artificial saliva component
Lentinan	Anticancer pharmaceutical Anti-HIV and anti-hepatitis pharmaceutical Malignant pleural effusion treatment Nutraceutical with immunomodulation activity
Grifolan	Nutraceutical with immunostimulation activity (indirect antitumor activity)
Schizophyllan	Nutraceutical with immunomodulation activity (indirect antitumor activity) Cosmetic ingredient with soothing/anti-inflammatory properties
Krestin	Nutraceutical with immunomodulation activity (indirect antitumor activity)
Reishi polysaccharide	Nutraceutical with immunomodulation activity (indirect antitumor activity) Several nutraceutical and pharmaceutical uses
**Lichen origin**	
Lichenan	Scientific research (bioactivity and enzyme activity)
Pustulan	Scientific research (bioactivity and enzyme activity)

^1^ Claim with insufficient scientific evidence; ^2^ Also extractable from bacteria; ^3^ Also extractable from fungi.

**Table 5:** Polysacchrides used in markets and their potential values.

Polysaccharides	Potential Market Value in USD/Year	Year	Source
Hyaluronic acid	9.6 billions	2020	https://www.reportsanddata.com/report-detail/hyaluronic-acid-market (accessed on 4 September 2021).
Heparin	6.5 billions	2020	https://www.alliedmarketresearch.com/heparin-market-A06186 (accessed on 4 September 2021).
Chitin/chitosan	4.2 billions	2021	https://www.bccresearch.com/market-research/plastics/chitin-chitosan-derivatives-markets-report.html (accessed on 4 September 2021).
Chondroitin sulfate	1.2 billions	2020	https://www.grandviewresearch.com/industry-analysis/chondroitin-sulfate-market (accessed on 4 September 2021).
Agar	239 millions	2020	https://www.industryarc.com/Research/Global-Agar-Market-Research-509553 (accessed on 4 September 2021).
Inulin	2.35 billions	2020	www.grandviewresearch.com/industry-analysis/inulin-market (accessed on 4 September 2021).
Pectin	888 millions	2020	https://www.mordorintelligence.com/industry-reports/pectin-market (accessed on 4 September 2021
Alginate	728 millions	2020	https://www.grandviewresearch.com/industry-analysis/alginate-market (accessed on 4 September 2021).
Carrageenan	742 millions	2019	https://www.grandviewresearch.com/industry-analysis/carrageenan-market (accessed on 4 September 2021).
xanthan	191 millions	2019	https://www.industryresearch.co/global-dextran-market-18478845 (accessed on 4 September 2021).
Cellulose	390 millions	2020	www.industryresearch.co/global-microbial-and-bacterial-cellulose-market-18720450 (accessed on 4 September 2021).
Pullulan	126 millions	2020	https://www.industryresearch.co/global-pullulan-market-18823725 (accessed on 4 September 2021).
Fucoidan	30 millions	2020	https://www.360researchreports.com/global-fucoidan-market-14030270 (accessed on 4 September 2021).
Laminarin	2 millions	2019	https://www.360researchreports.com/global-laminarin-market-18049714 (accessed on 4 September 2021).
Lentinan	10 millions	2019	www.360marketupdates.com/global-lentinan-market-14829124 (accessed on 4 September 2021).
